# The gut microbiome changes in wild type and IL-18 knockout mice after 9.0 Gy total body irradiation

**DOI:** 10.1186/s42523-023-00262-8

**Published:** 2023-09-07

**Authors:** Wanchang Cui, Lisa Hull, Alex Zizzo, Li Wang, Bin Lin, Min Zhai, Mang Xiao

**Affiliations:** 1 Armed Forces Radiobiology Research Institute, Uniformed Services University of the Health Sciences, 4555 South Palmer Road, Bethesda, MD 20889-5648 USA; 2 The Henry M. Jackson Foundation for the Advancement of Military Medicine, Inc., Bethesda, MD 20817 USA; 3 Department of Pharmacology and Molecular Therapeutics, Uniformed Services University of the Health Sciences, Bethesda, MD 20814 USA; 4 Department of Pathology, Uniformed Services University of the Health Sciences, Bethesda, MD 20814 USA

**Keywords:** Ionizing radiation, IL-18 knockout mice, Gut microbiome

## Abstract

**Background:**

Recent studies have shown that gut microbiome plays important roles in response to radiation exposure. IL-18, an inflammatory cytokine, is highly elevated in mice, mini-pigs and nonhuman primates after radiation exposure. Blocking IL-18 using its endogenous binding protein (IL-18BP) increases mice survival after radiation exposure by decreasing bone marrow interferon-gamma levels.

**Methods:**

To further characterize the roles of IL-18 in response to radiation, both wild type and IL-18 knockout (IL-18 KO) mice were exposed to 9.0 Gy total body irradiation (TBI). The 30-day survival result demonstrated that IL-18 KO mice were significantly more resistant to radiation compared to the wild type mice (p < 0.0001). Mouse faecal samples were collected at pre-radiation (d0), d1, d3, d7, d14, d21 and d29 after radiation exposure. Microbiome profiling was performed on the faecal samples using 16S and ITS sequencing technology.

**Results:**

Data analysis showed that there was significant difference in the bacterial microbiome between wild type and IL-18 KO mice. Cohousing of wild type and IL-18 KO mice decreased the bacterial microbiome difference between the two genotypes. Much fewer bacterial genera were significantly changed in wild type mice than the IL-18 KO mice after radiation exposure. The different composition of the IL-18 KO mice and wild type mice persisted even after radiation exposure. Bacterial genera that significantly correlated with other genera were identified in the IL-18 KO and wild type mice. The metabolic pathways that differentially expressed in both genotypes were identified. The animal bacterial microbiome data could be used to predict the animal’s radiation status. The fungal microbiome had no significant difference regarding genotype or time after radiation exposure.

**Conclusion:**

The current study helps understand the gut microbiome in different genetic backgrounds and its temporal changes after radiation exposure. Our data provide insight into the mechanisms underlying radiation-induced toxicity and help identify bacteria important in response to radiation.

**Supplementary Information:**

The online version contains supplementary material available at 10.1186/s42523-023-00262-8.

## Background

Radiation is a serious health threat to the general public or first responders in the event of nuclear accidents or intended terrorist attacks [1, 2]. Exposure to ionizing radiation (IR) is associated with the induction of acute radiation syndromes (ARS); the hematopoietic (H-) and gastrointestinal (GI) are the two systems mostly affected after high dose radiation exposure [3]. Currently four FDA-approved drugs are available for the treatment of H-ARS (including Neupogen, Neulasta, Leukine and Nplate) [4]. However, these mitigators have limited efficacy (increased survival by ~ 30%) and radiation is known to cause multiple organ injuries. Novel approaches or radiation mitigators are still urgently needed, particularly for non-H-ARS.

Microbiome or microbiota reflects the whole collection of microorganisms living in a specific environment, such as that of human or animal bodies. It was estimated that there were actually a larger number of bacteria than that of human cells in a “reference” man [5]. Microbiome has been shown to play very important roles in different physiological conditions and diseases such as metabolic diseases, cancer, etc. Recent studies showed that radiation and the microbiome are tightly connected. Guo et al. [6] suggested that mice that survived lethal doses of radiation, so called “elite survivors”, had distinct gut microbiome composition. *Lachnospiraceae* and *Enterococcaceae*, together with downstream metabolites represented by propionate and tryptophan pathway members, contributed substantially to radioprotection. Cui et al. showed that same mouse species intestinal microbe transplantation could alleviate radiation-induced toxicity in a sex-dependent manner [7]. Furthermore, it was shown that valeric acid, a gut commensal derived metabolite, significantly increased mouse survival after radiation [8]. Taken together, the literature suggests that radiation significantly affected the gut microbiome and modifying the gut microbiome could potentially positively affect the hosts’ response to radiation toxicity.

IL-18 is a pro-inflammatory cytokine in the family of IL-1, playing important roles in inflammatory and immune responses [9]. IL-18 is produced as a pro-protein, which is cleaved by caspase-1. The active IL-18 binds to its receptor IL-18Rα and then recruits IL-18Rβ to form a high affinity signalling complex. The complex is recognized by Toll-like receptors to initiate inflammatory signal transduction. IL-18 binding protein (IL-18BP) is a natural antagonist of IL-18. Under normal conditions, excessive amounts of IL-18BP will neutralize IL-18 levels thus controlling its activity. Our published data showed that IL-18 played important roles in radiation responses. IL-18 is persistently elevated in mouse, minipig, and NHP serum and/or urine in a radiation dose and time-dependent manner [10, 11]. Data from our recent report demonstrated that IL-18BP treatment inhibited IL-18 signalling and decreased the expression of IL-18’s downstream target interferon (IFN)-γ in mouse bone marrow and significantly increased survival of mice from 30% to 55% after total body irradiation (TBI) [12].

In this study, we aimed to further investigate the roles of IL-18 in response to radiation using IL-18 knockout mice. IL-18 knockout and wild type mice were exposed to a lethal dose of radiation for survival studies. Their faecal pellets were collected for microbiome analysis. We expected to study the microbiome composition of the wildtype and IL-18 knockout mice, before and after radiation exposure, and to identify bacteria and fungi that play important roles in the host’s response to radiation exposure.

## Results

### IL-18 is a determining factor in mouse survival after TBI

**Fig. 1:**
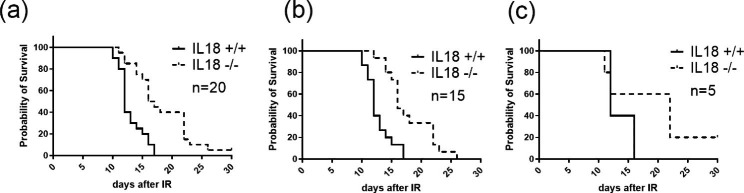
Survival curves of IL-18 knockout and wild type mice after 9.0 Gy TBI. (**a**) the survival curves of all the mice (n = 20 mice/genotype), (**b**) the survival curves of the purely housed mice only (n = 15 mice/genotype), (**c**) the survival curves of the mixed mice only (n = 5 mice/genotype). Thirty-day survival was monitored after gamma radiation exposure

First, we performed survival studies of IL-18 knockout mice and wild mice after 9.0 Gy TBI. The survival study was designed using a one-tailed, 5% significance level, 80% power, allocation ratio of 1, to detect a 40% difference in mortality (i.e. 70% mortality vs. 30% mortality). Based on the power analysis, the sample size (animal number in each group) was 20 mice per group. 20 wild type and 20 IL-18 knockout female mice were used for the survival study. Before irradiation, 15 mice of each genotype were housed with the same genotype peers in the same cage (which were called “pure mice”) and 5 mice of each genotype were accidently mixed with their opposite genotype (which were called “mixed mice”). Mice were housed in our vivarium for two weeks before irradiation. Each mouse was individually tracked for its survival and faecal pellet collection. Survival data in Fig. 1 showed that IL-18 knockout mice were significantly more resistant to radiation than wild type control mice. Figure 1a showed the survival curves of all the 20 IL-18 knockout mice and 20 wild type mice after 9.0 Gy TBI. Figure 1b showed the survival curves of “pure” IL-18 knockout and wild type mice only (n = 15 each). Figure 1c showed the survival curves of the mixed IL-18 knockout and wild type mice only (n = 5 each). For all the mice in the study (n = 20), the median survival was 12 days for the wild type mice and 16.5 days for the IL-18 knockout mice; the survival difference was significant between the two genotypes (p = 0.0002). For the pure mice only (n = 15), the median survival was 12 days for the wild type mice and 16 days for the IL-18 knockout mice; the survival difference was significant between the two genotypes (p = 0.0002). For the mixed mice (n = 5), the median survival was 12 days for the wild type mice and 22 days for the IL-18 knockout mice; the survival difference was not significant between the genotypes.

### 16S rRNA sequencing sample information and read statistics

The numbers of collected faecal pellets were shown in Supplementary Table S1. All the collected faecal samples (186 samples) were used for microbiome sequencing. Read counts of all sequences varied from 13,889 to 59,807 with a median frequency of 33521.5, GC% of 52–55. Dups% of 79.80 to 97.10 (shown in Supplementary Table S2). The FastQC sequence count, mean quality score, per sequence quality score of each sample were shown in Supplementary Figs. S1-S3 and Supplementary Table S2. The alpha rarefaction of forward sequencing showed that all samples had sufficient coverage which plateaued at 5,000 reads (Supplementary Fig. S4).

### Genotype determines the faecal bacterial microbiome and co-housing decrease the faecal bacterial microbiome difference between the two genotypes

**Fig. 2:**
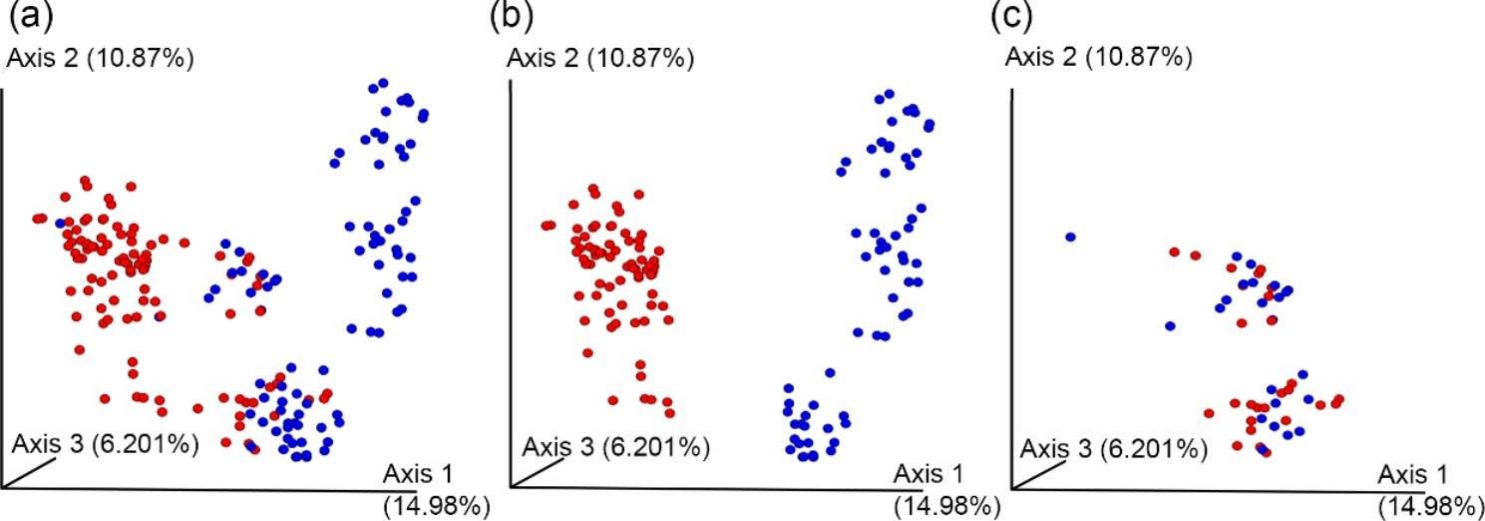
The PCOA plot showing the differences in the faecal microbiome between the IL-18 knockout and wild type mice based on 16S rRNA sequencing. (**a**) the PCOA plot of all mice (n = 20 mice/genotype), (**b**) the PCOA plot of purely housed mice only (n = 15 mice/genotype), (**c**) the PCOA plot of the mixed mice only (n = 5 mice/genotype). Each dot represents one faecal sample of one mouse at one time. Red, IL-18 knockout mouse; blue, IL-18 wild type mouse

Principal Coordinates Analysis (PCOA) is often used to find characteristic patterns associated with certain factors such as genotype, age, treatment, etc. We performed PCOA on the gut bacterial microbiome of the wild type and IL-18 knockout mice before and after irradiation (Fig. 2). The genotype could not completely separate the wild type and IL-18 knockout mice when all the mice were considered (Fig. 2a). However, if only the “pure mice” were considered, there was a clear separation between these two genotypes (Fig. 2b). If only the “mixed mice” were considered, these two genotypes were completely mixed. Other factors such as radiation, time after irradiation, etc., could not have a clear separation between groups (data not shown). The PCOA data suggest that the IL-18 genotype had a determining effect on the gut microbiome composition. Co-housing IL-18 knockout and wild type mice for 2 weeks decreased the gut microbiome difference between these two genotypes. It is well known that mice are coprophagic animals, which explains the co-housing effect on gut microbiome of IL-18 knockout and wild type mice. Because of the co-housing effect, all the remaining analysis were done using the “pure mice” only unless specifically noted.

### Alpha and beta diversity of the pure IL-18 knockout and wild type mice

**Fig. 3:**
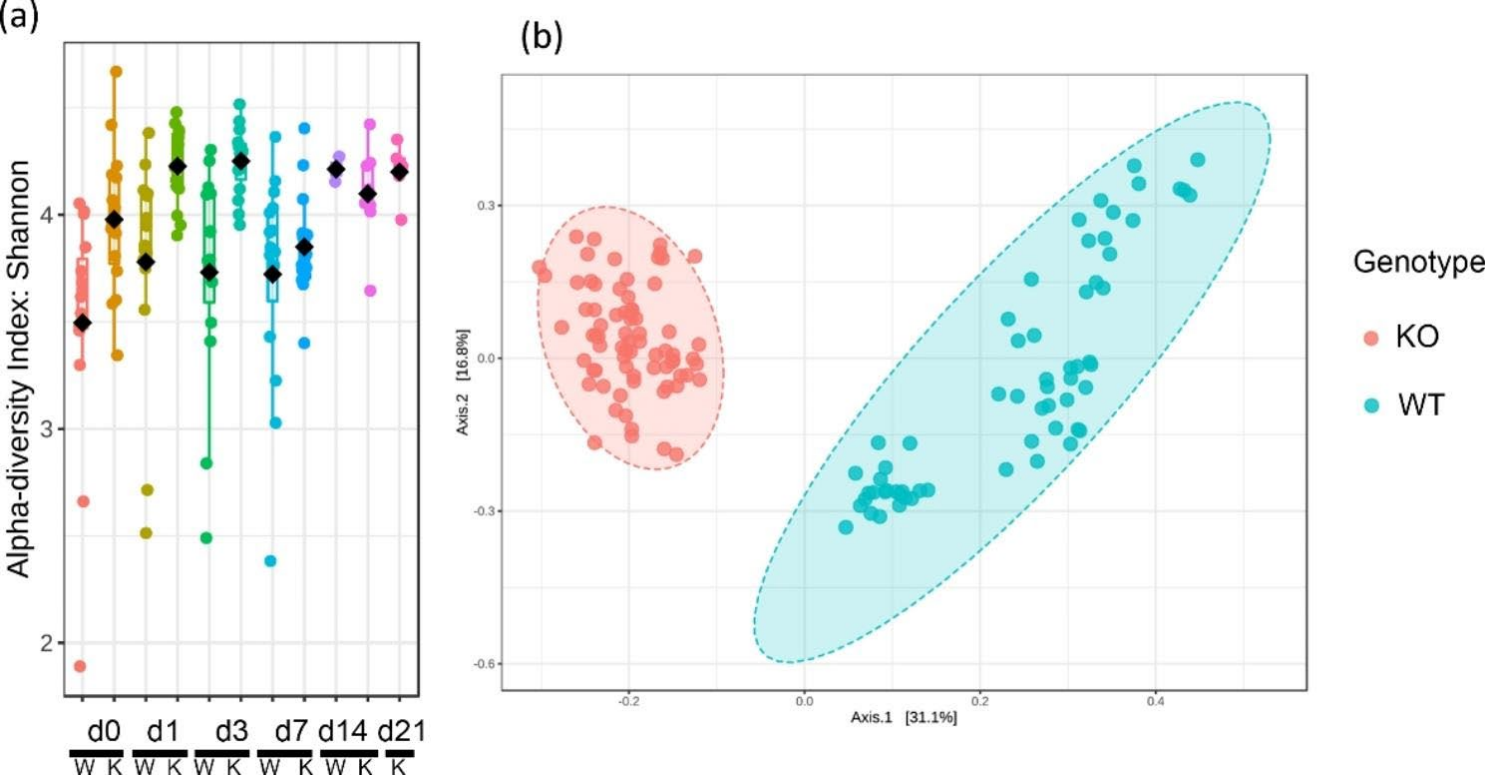
The alpha and beta diversity of the pure IL-18 knockout and wild type mice faecal bacterial microbiome. Alpha-diversity, measured by observed species and Shannon Diversity Index is plotted for IL-18 knockout and wild type mice at different time points after radiation exposure. The black dot inside the box represents the median, while the whiskers represent the lowest and highest values within the 1.5 interquartile range (IQR). Outlines as well as individual sample values are shown as dots. (**b**) beta diversity showing the complete separation of the pure IL-18 knockout and wild type mice. Each dot represents one animal at one time point

Alpha and beta diversity are two parameters frequently used in microbiome analysis. Alpha diversity measures the variation in a single sample while beta diversity measures the variation of microbial communities between samples. As shown in Fig. 3a, the IL-18 knockout mice had a higher alpha diversity than the wild type mice at multiple time points after irradiation (d0, d1, d3, d7). As shown in Fig. 3b, there was clear separation of the IL-18 knockout and wild type mice based on the beta diversity, which is similar to the PCOA result of Fig. 2b. Other factors such as radiation, time after irradiation, could not separate the animals based on the beta diversity (data not shown).

### More significantly changed bacterial genera in the IL-18 knockout mice than wild type mice after irradiation

**Fig. 4:**
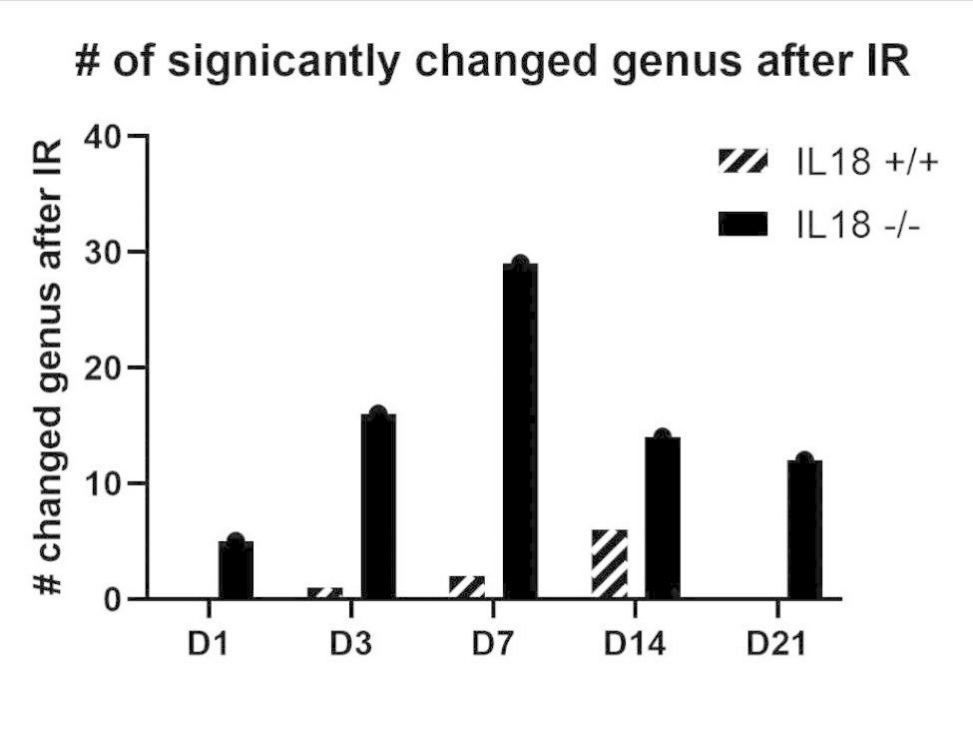
The significantly changed number of bacterial genera of the pure IL-18 knockout and wild type mice after irradiation compared to their un-irradiated samples respectively

We quantified the numbers of bacterial genera that were significantly different after irradiation compared to pre-irradiation in IL-18 knockout and wild type mice respectively. There were more significantly changed bacterial genera in the IL-18 knockout mice compared to wild type mice (Fig. 4). There were 0, 1, 2, 6 significantly changed genera in the wild type mice at day 1, 3, 7 and 14 after irradiation; while there were 5, 16, 29, 14 and 12 significantly changed genera in the IL-18 knockout mice at day 1, 3, 7, 14 and 21 after irradiation. The significantly changed genera in each genotype after irradiation were listed in Supplementary Table S3.

### The bacterial microbiome difference between the pure IL18 knockout and wild type mice shown by heatmap

**Fig. 5:**
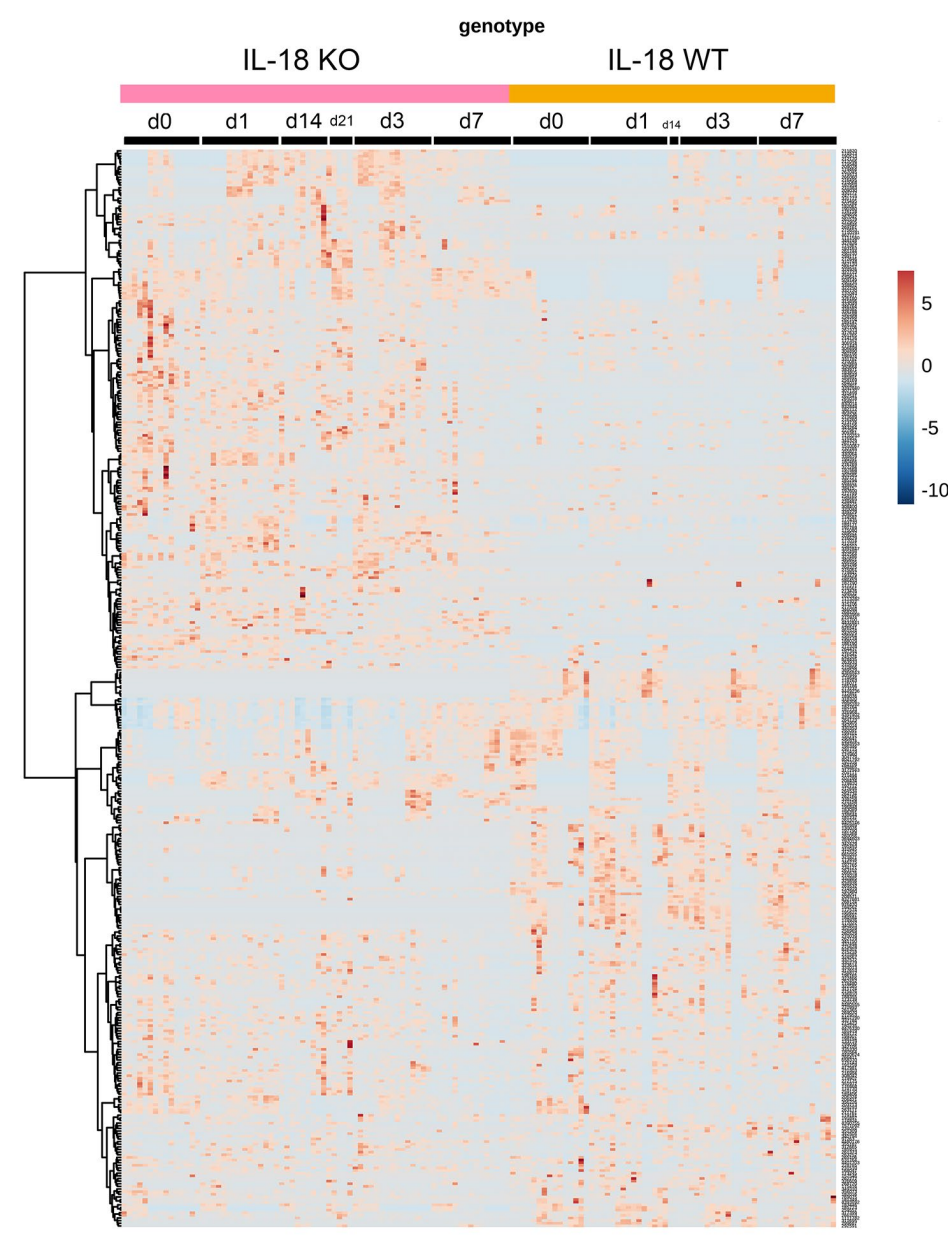
The heatmap of faecal bacterial microbiome of pure IL-18 knockout and wild type mice. Each column represents one faecal sample and each row represents one microbiome feature. Blue represents lower level of expression and brown represents higher level of expression

A Heatmap is frequently used to compare differentially expressed targets by clustering analysis. The Heatmap in Fig. 5 showed that the IL-18 knockout and wild type mice had differential expression of their faecal microbiome. The top clustered microbiome features were highly expressed in the IL-18 knockout mice while the bottom clustered microbiome features were highly expressed in the IL-18 wild type mice.

### The difference of bacterial microbiome composition between IL-18 knockout and wild type mice

**Fig. 6:**
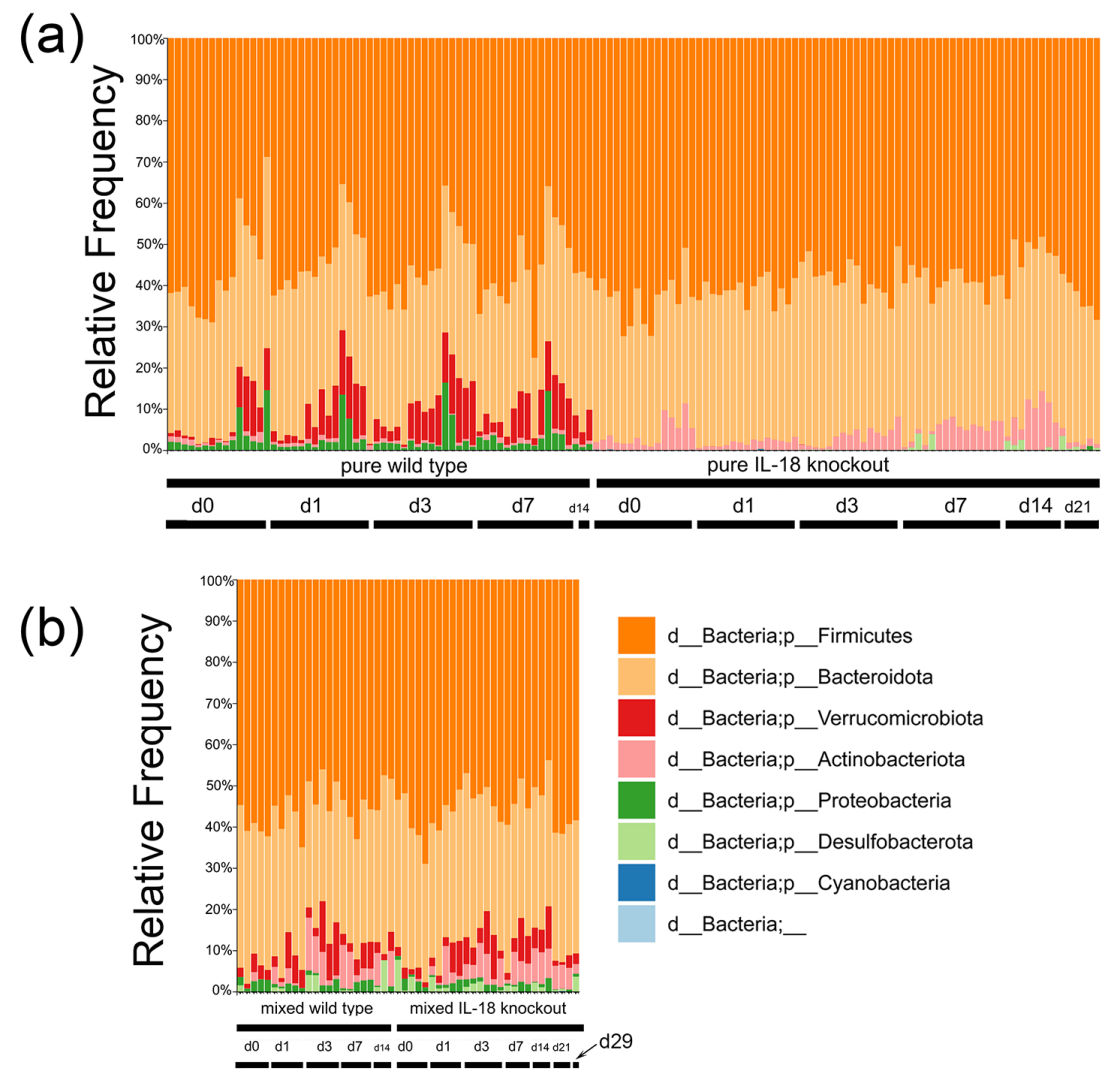
The relative abundance of bacterial microbiome composition at the phylum level of pure and mixed IL-18 knockout and wild type mice at different time points after radiation exposure. (**a**) pure mice only. (**b**) mixed mice only. The seven phyla are shown on the right (same for both (**a**) and (**b**)). Each vertical bar represents one faecal sample. Genotype, cohousing status and time after irradiation were labelled at the bottom

We studied the differences in the faecal bacterial microbiome of the IL-18 knockout and wild type mice before and after irradiation. Figure 6 showed there were very different compositions of the pure wild type and knockout mice at the phylum level. *p-Firmicutes* and *p_Bacteriodota* were the most abundant phyla in both genotypes. However, there was a high level of *p_Actinobacteriota* in the IL-18 knockout mice at multiple time points but its levels was low in the wild type mice. The levels of *p_Verrucomicrobiota* and *p_Proteobacteria* were very high in the wild type mice but very low in the IL-18 knockout mice. Co-housing blended the levels of *p_Actinobacteriota*, *p_Verrucomicrobiota* and *p_Proteobacteria* in the mixed wild type and knockout mice. Another observation was that *p_Desulfobacterota*, which was rarely seen in the pure wild type and knockout mice, had a higher presence in the mixed wild type and knockout mice. The results suggested that there were fundamental differences in the faecal bacterial microbiome of the IL-18 knockout mice and wild type mice, and the difference persisted even after radiation exposure. Cohousing of wild type and knockout mice changed their microbiome composition.

### Identifying differentially expressed bacteria on the phylum, genus, and species levels in both genotypes after irradiation

**Fig. 7:**
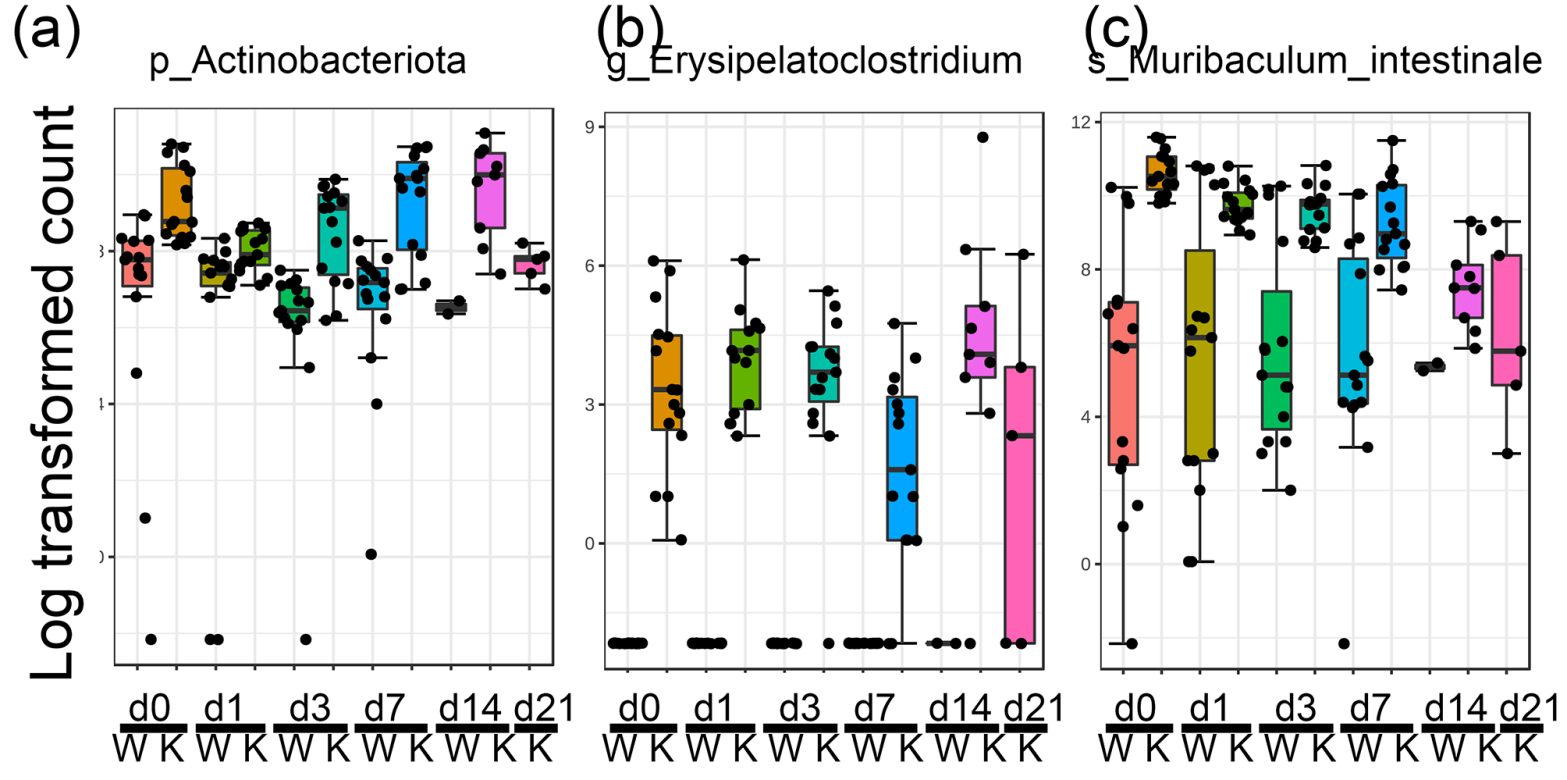
Examples of the significantly different bacteria at the phylum, genus and species level between the IL-18 knockout and wild type pure mice at different time points. W, wild type; K, knockout

Using classical univariate statistical comparisons, we identified the differentially expressed bacteria across the genotype/radiation groups on the phylum, genus and species levels. There were 3, 42 and 24 differentially expressed bacterial phyla, genera and species between pure IL-18 knockout and wild type mice, respectively. Figure 7 showed a graph of an example of the differentially expressed bacteria at different taxa levels. *p_Actinobacteriota*, *g_Erysipelatoclostriudim*, *s_Murbaculum_intestinale* had significantly higher abundance in the IL-18 knockout mice compared to the wild type mice at multiple time points. All the significantly changed bacteria at the phylum, genus and species levels were listed in Supplementary Table S4.

### Identifying bacteria that significantly correlated with other bacteria in the IL-18 knockout and wild type mice

**Fig. 8:**
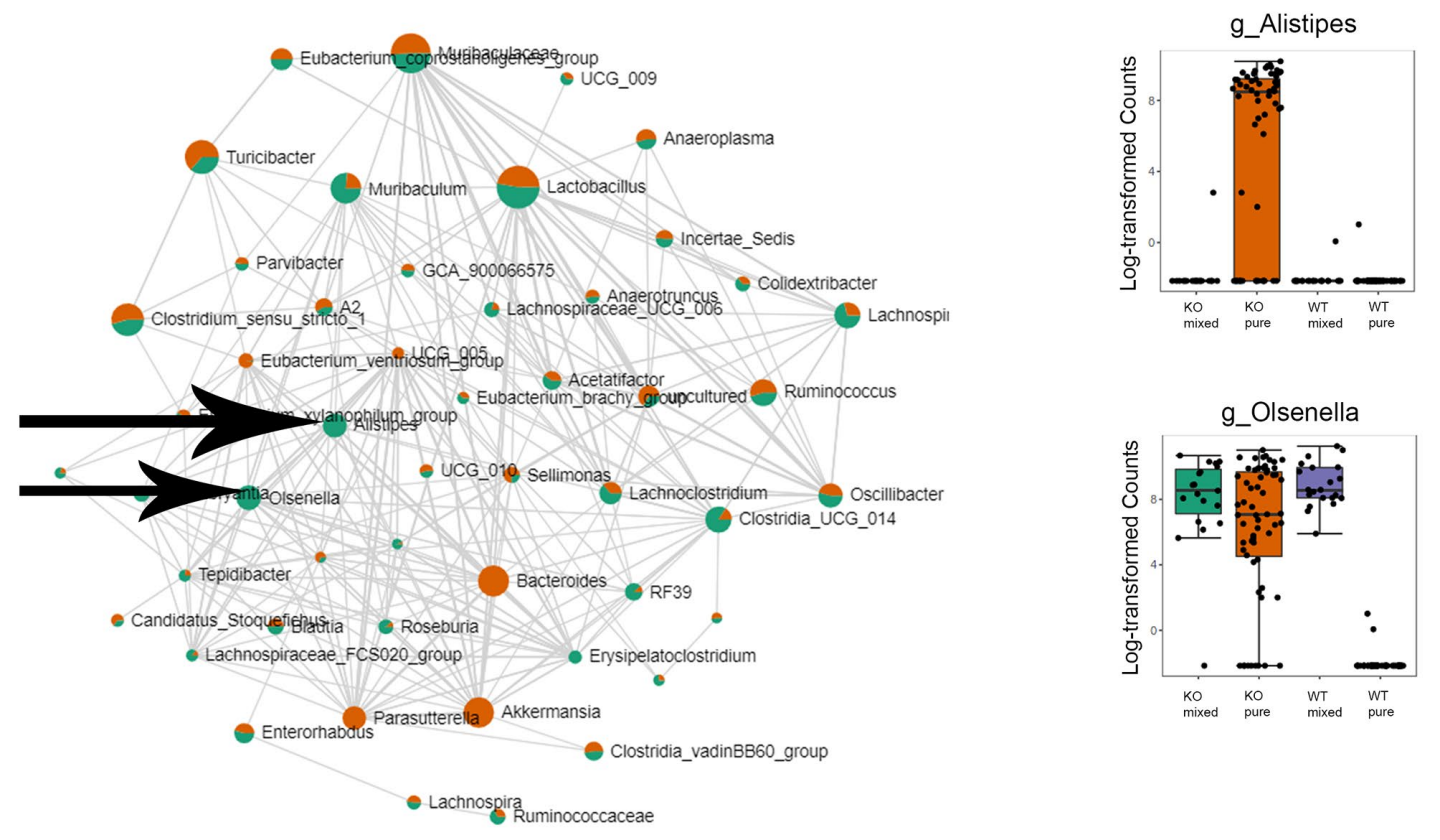
The correlation map of the significantly correlated bacterial genera based on the microbiome data in the pure IL-18 knockout and wild type mice. Two of the genera that have high expression in the IL-18 knockout mice but low expression in the wild type mice are labelled with black arrows. Their relative expression levels are plotted on the right

Bacteria that significantly correlate with other bacteria potentially have more significant roles in the microbiome. Figure 8 showed the correlation map and two bacteria genera examples. There were about 500 significantly correlated pairs between the bacteria genera in this study (Supplementary Table S5). *g_Alistipes* and *g_Olsenella* were the two bacteria genera that had significant correlations with many other bacteria and also highly expressed in the IL-18 knockout mice but with very low expression in the wild type mice.

### Predictive functional profilling of microbial communities in the IL-18 knockout and wild type mice

**Fig. 9:**
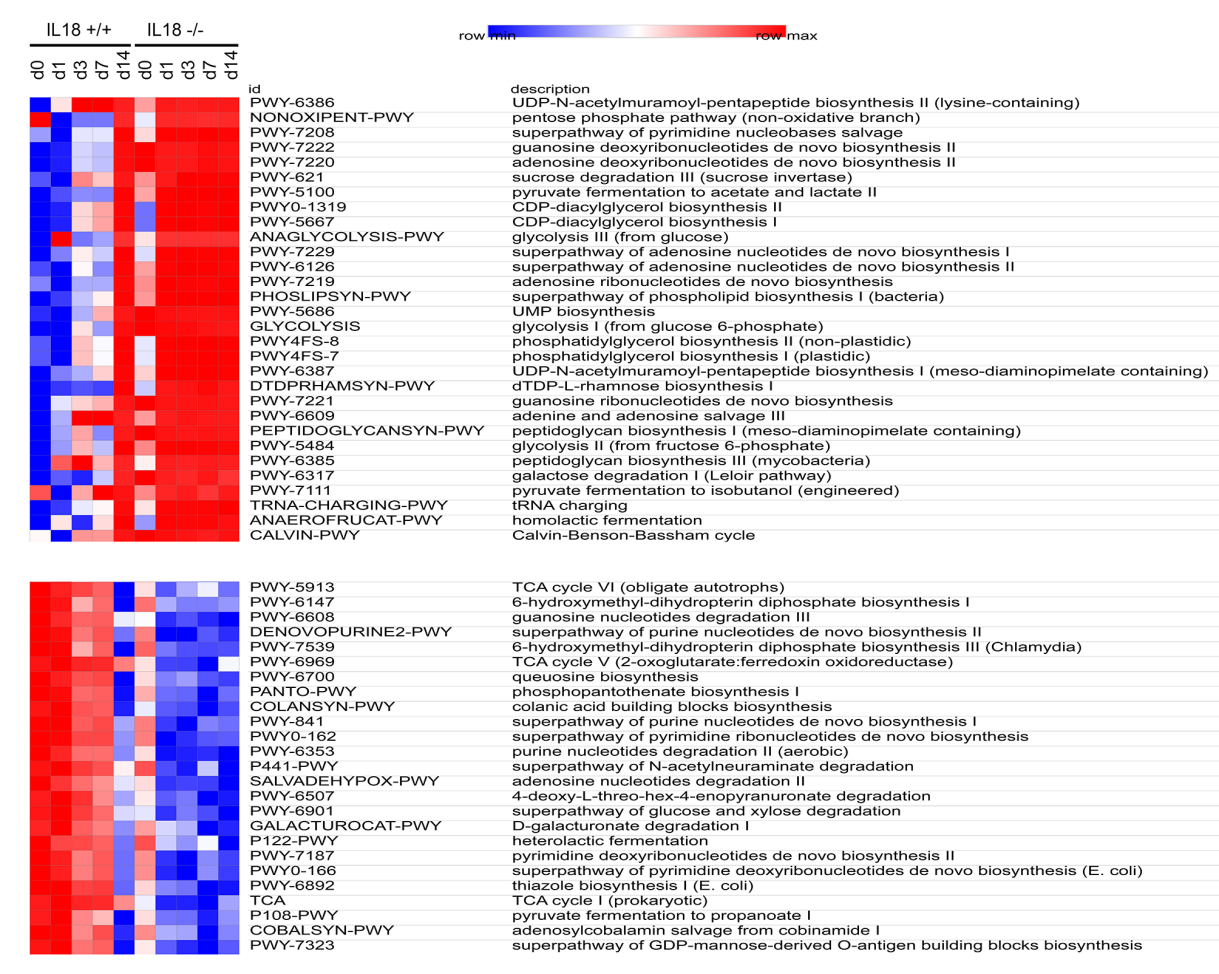
The different metabolic pathways between the IL-18 knockout and wild type mice predicted by CITRUS2 method. Each column represents one group of mice, each row represents one metabolic pathway id and its description

Phylogenetic investigation of communities by reconstruction of unobserved states (PICRUSt) is a computational approach to predict the functional composition of a metagenome using marker gene data and a database of reference genomes. Using the PICRUST2 pipeline, we identified significantly different metabolic pathways in the IL-18 knockout and wild type mice based on the bacterial microbiome data (an excerpt of the metabolic pathways was shown in Fig. 9 and the full image of the metabolic pathways was shown in Supplementary File S5). There were large increases in the amino acid synthesis and metabolism pathways (particularly in those associated with valine, L-isoleucine, etc. ) and in nucleic acids (particularly in pathways associated with adenosine) in the IL-18 knockout mice.

### Using bacterial microbiome data to estimate the radiation exposure

**Fig. 10:**
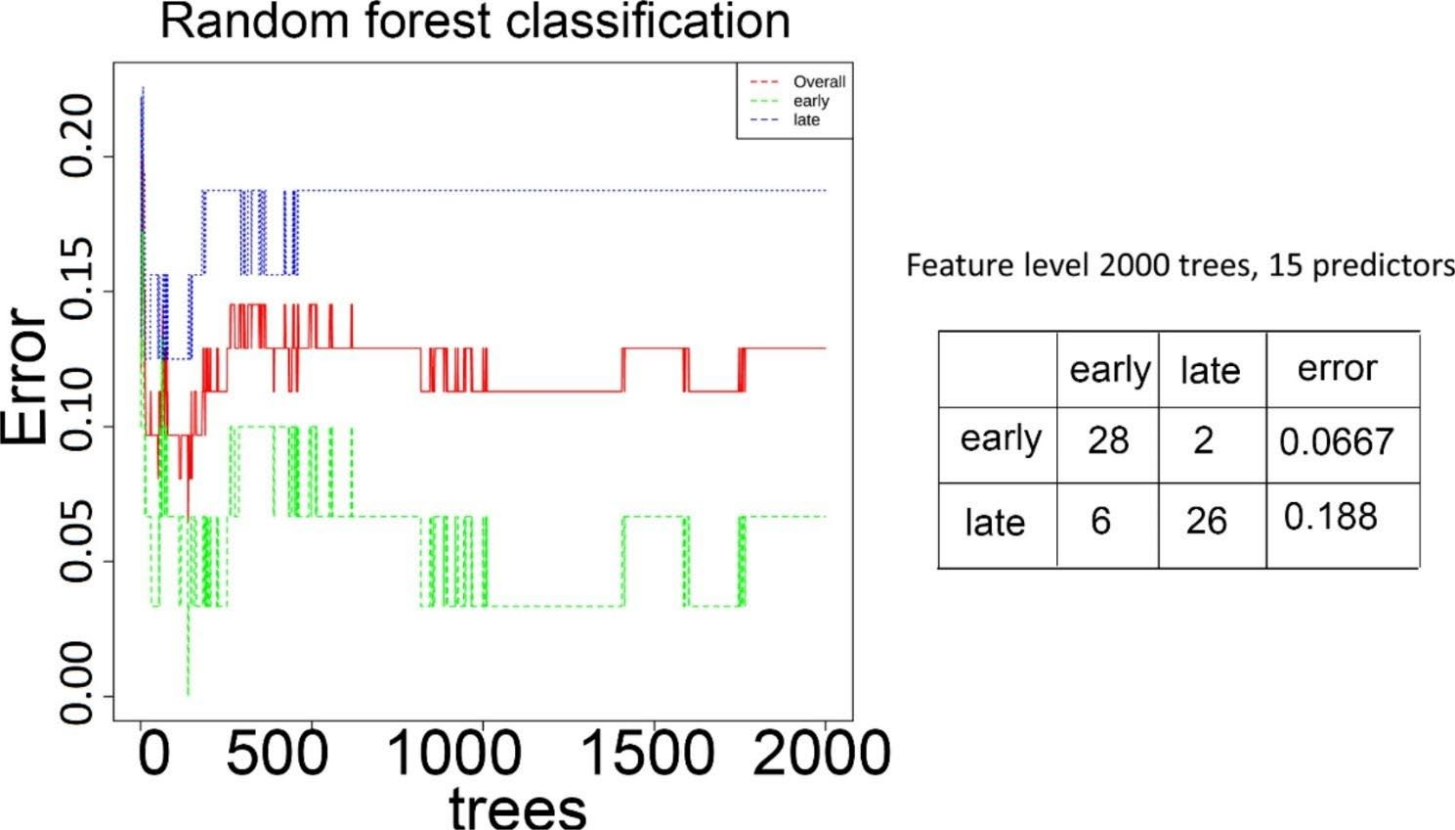
The random forest classification of early samples vs. late samples. After 2000 trees, with 15 predictors, the algorithm can predict whether one sample is in the early group or late group

Microbiome data has been used to predict pathological or physiological conditions. We could not estimate the radiation dose using the current dataset since all these animals were exposed to one radiation dose only. Here, we estimated the time after radiation exposure of each animal in the wild type mice only. Since we know that there was no significantly changed bacteria on d1 compared with the baseline, we separated the wild type mice into two groups, early group (d0 and d1 samples) and late group (d3, d7, and d14). Using a random forest tree method, we estimated the possibility that each animal belonged to which group (Fig. 10). Out of the 30 early samples, we correctly identified 28 of them; out of the 32 late samples, we correctly identified 26 of them. The data suggest that microbiome data can be used to estimate the host’s radiation status with low errors.

### No significant change in the fungal microbiome in the IL-18 knockout and wild type mice before and after radiation exposure

We also studied the fungal microbiome of the IL-18 knockout and wild type mice before and after radiation exposure using the same PCOA analysis and there was no separation of the animals based on genotypes, cohousing status, time after radiation exposure (Supplementary Fig. S6). We also studied the relative abundance between the different genotype and time after radiation exposure groups, and there were no significantly different fungi between any groups (data not shown). The data suggest that there were no significant changes of the fungal microbiome in the IL-18 knockout and wild type mice before and after radiation exposure.

## Discussions

### Genotype plays a more important role in the faceal microbiome composition than external factors, such as a lethal dose of radiation

Our data showed that IL-18 knockout mice had a distinct bacterial microbiome composition compared to the wild type mice, and the difference persisted even after a lethal dose of radiation exposure. Our data are in agreement with literature suggesting that mouse genetics have a more dominant role in shaping the gut microbiome than the mouse’s environment [13]. Similar to IL-18, at least 30 specific genes have been identified to have specific effects on the microbiome using genetically engineered knockout mice, suggesting that the microbial composition is at least partially regulated by the host genetics [14]. IL-18 is known to regulate the host microbiota. For example, it has been shown that IL-18 induced anti-microbial peptide (AMP) secretion, therefore controlling the microbiota colonization in the wild type mice [15]. It is reasonable to hypothesize that in the IL-18 knockout mice, there is lower levels of AMP production, thus some bacterial genera that had lower expression in the wild type mice, had elevated levels in the IL-18 knockout mice (such as *g_Alistipes* and *g_Olsenella*). The current study identified *g_Alistipes* and *g_Olsenella* as two bacteria genera that had significant correlations with other bacteria and were also highly expressed in the IL-18 knockout mice but showed very low expression in the wild type mice. Alistipes is a known producer of single chain fatty acids (SCFAs) such as acetate and propionate [16, 17]. Acetate and propionate were shown to be highly elevated in the “elite survivors” after irradiation and had radioprotective effect [6]. Therefore these two bacteria genera may have pivotal roles in the IL-18 knockout mice’s resistance to radiation and they could be potential effective radiation mitigators. Instead of transplanting faecal samples, mice treated with a mixture of these two important bacteria genera may survive better after lethal doses of radiation.

Radiation is known to induce inflammasome pathway activation and inflammasome activation causes the maturation of pro-inflammatory cytokines such as IL-18 [18]. Recently it has been shown that inflammasome interacts with the gut microbiota in a reciprocal way through IL-18. The gut microbiota can induce the expression and activation of inflammasome proteins, and also inflammasome activation can modulate the composition of gut microbiota [19]. The deletion of IL-18 in mice may intercept the deleterious effects of inflammasome overactivation and gut microbiota change, thus decreasing mouse mortality after lethal radiation exposure.

Our data showed that co-housing decreased the bacterial microbiome difference between the IL-18 knockout and wild type mice, supporting the idea that co-housing or faecal microbiota transplant (FMT) is an effective approach to change the microbiome composition. Co-housing and FMT have been shown to significantly improve mouse survival after lethal doses of radiation [6, 7]. However, cohousing or FMT’s effects on the recipients’ microbiota may be complicated, as shown in Fig. 8 that the levels of *g_Olsenella* were elevated in the wild type recipient mice through co-housing; however, the levels of *g_Alistipes* was only high in the pure IL-18 knockout mice, suggesting that the wild type mice may have some inhibitory effect on the colonization of *g_Alistipes* even in the IL-18 knockout recipient mice.

It is known that ionizing radiation can cause the gut micrombiota dysbiosis, increasing pathogenic bacteria abundance and decreaseing beneficial bacteria abundance [20]. In our pure wild type mice, *Muribaculaceae*, *Robinsoniella*, *Erysipelatoclostridium*, *Dickeya*, and *Candidatus_Stoquefichus* were signficantly elevated, while *[Eubacterium]_siraeum_group* and *Staphylococcus* were signficanlty decreased after 9.0 Gy radiation exposure. Their roles related to iozining radiation need to be studied in the future.

### Faecal microbiome have the potential to be a radiation biodosimetry technique

Gut microbome has been shown to reflect many human diseases and also their severity [21, 22]. Our data showed that the faecal microbiome data could be used to distinguish the early time points and late time points of the irradiated animals (Fig. 10). Similar to a number of “omics” approaches such as genomics, transcriptomics, proteomics, lipidomics and metaboblomics that have been investigated as radiation biodosimetry tools [23], we foresee the microbiome data can be used as a tool for radiation biodosimetry. Faecal sample has its own advantages, for example, it can be acquired noninvasively without special medical training, and processed in high-output manner. Since our data here used one radiation dose only, more work using different radiation doses are needed to establish the usefulness of faecal microbiome as a radiation biodosimetry tool.

### Potential implications of using *Alistipes* supplements for human health

Persistent gut microbial dysbiosis are known to be associated with radiotherapy or chemotherapy side effects [24, 25]. FMT has shown great promise to not only mitigate chemo- or radiation side effects, but also increase the efficacy of radiotherapy or chemotherapy [25, 26]. However, FMT can be associated with life-threatening adverse events due to transmission of pathogenic organisms such as enteropathogenic Escherichia coli (EPEC) and Shigatoxin-producing Escherichia coli (STEC) from stool banks. Therefore, preparation of pure beneficial bacteria will be needed for clinical use instead of using faecal materials. *Alistpes* may be one bacteria genus of interest to mitigate radiation and chemotherapy toxicity based on literature and our results here. It has been shown that *Alistpes* was substantially decreased during chemotherapy in acute lymphoblastic leukemia patients [27]. FMT has been shown to significantly alleviate radiaton-induced intestinal edema, where *Alistpes* might play important roles [28]. Our data here identified *Alistepes* highly abundant in the radiation-resistant mice compared to more sensitive mice. Although the current understanding is still preliminary, future work may prove the usefulness of *Alistpes* for this purpose.

## Conclusions

In summary, our data showed that IL-18 knockout mice had a distinct faecal microbiome than the wild type mice, and the difference persisted even after a lethal dose radiation exposure. The difference in the IL-18 knockout and wild type mice may help to explain the different radiation sensitivity of the IL-18 knockout and wild type mice. We identified potentially important bacterial genera which may play important roles in radiation sensitivity and potentially be developed as radiation mitigators. The faecal microbiome showed potential as a radiation biodosimetry tool.

## Materials and methods

### Ethics statement

Animals were housed in an Association for Assessment and accreditation of Laboratory Animal Care (AAALAC)-approved facility at the Uniformed Services University of the Health Sciences (USUHS). All animal study procedures including housing, irradiation, survival study, and blood/tissue collection were reviewed and approved by the USUHS Institutional Animal Care and Use Committee (IACUC) and all experiments were performed in accordance with guidelines and regulations from the USUHS-IACUC and the USUHS Department of Laboratory Animal Resources (DLAR).

### Mice and animal care

Twelve- to 14-week-old female C57BL6 IL-18 wild type and knockout mice (Jackson Laboratory, Bar Harbour, ME) were used. Mice were randomly housed in an AAALAC-approved facility at the USUHS. Animal rooms were maintained at 20–26 °C with 30–70% humidity on a 12 h light/dark cycle. Commercial rodent chow (Harlan Teklad Rodent Diet 8604) was available *ad libitum* as was acidified water (pH = 2.5–3.0) to control opportunistic infections. PCR genotyping was performed to confirm IL-18 knockout or wild type status of each mouse.

### Irradiation

Mice received TBI in a bilateral radiation field at the Armed Forces Radiobiology Research Institute (AFRRI)’s ^60^Co facility. The alanine/electron spin resonance (ESR) dosimetry system (American Society for Testing and Materials, Standard E 1607) was used to measure dose rates (to water) in the cores of acrylic mouse phantoms. The midline tissue dose to the mice was 9.0 Gy at a dose rate of 0.6 Gy/min. The day of irradiation was considered day 0.

### Thirty‑day survival study and faecal pellet collection

Mice were observed for 30 days to determine the survivability after 9.0 Gy (LD_90/30_) TBI exposure (N = 20 mice/group). Animals were euthanized when they met predetermined criteria before the end of study [29]. Mouse faecal pellets were collected pre-irradiation (d0), d1, d3, d7, d14, d21 and d29 after irradiation from all alive animals. Fresh mouse faecal pellets were stored at -80^o^C until analysis.

### Sequencing of the gut microbiome

The DNA extraction, generation of amplicons, and sequencing were performed by the University of Minnesota Genomics Center. Briefly, faecal DNA was extracted using a DNeasy PowerSoil Pro Kit (Qiagen, Hilden Germany), followed by amplification of the V3-V4 region of the 16S rRNA and internal transcribed spacer 1 (ITS1) gene using standard methods [30]. DNA libraries were generated from the resulting amplicons using the Illumina TruSeq Nano kit (Illumina, San Diego, CA), and amplicons were then sequenced by the Illumina MiSeq platform using the 2- by 300-bp paired-end V3V4 and ITS1 kit (Illumina).

### 16S rRNA sequencing analysis

16S Sequences were pre-processed, quality filtered, and analysed using the Nephele platform (https://nephele.niaid.nih.gov/) from the National Institute of Allergy and Infectious Diseases (NIAID) Office of Cyber Infrastructure and Computational Biology (OCICB) in Bethesda, Maryland, USA [31]. Briefly, the QIMME2 pipeline in Nephele was used to analyse samples with default settings. The biom files generated from the QIIMME2 pipeline were used for Nephele downstream analysis and further analysis in MicrobiomeDB (www.microbiomedb.org) and MicrobiomeAnalyst (www.microbiomeanalyst.ca). The Nephele, microbiomeDB and MicrobiomeAnalyist analysis were performed using the default parameters. Specifically, for the Nephele pre-process step, the FastQC sequence quality check and Summary graphs of QC steps were selected. For the Nephele Analyze step, QIMME2 2.0 16S pipeline was used for 16S data analysis. Paired End FASTQ was selected for data import, and 25 for Minimum Phred Quality Score, Closed reference for clustering algorithm, 97 for OTU reference, mafft for Phylogenetic tree, sklearn for Taxonomy classification method, SILVA V4 region for sklearn options, 2000 for Barplots Filter Samples Minimum Frequency, and 0.97 for Perc Identify. For the Explore: Downstream Analyses: Diversity, the BIOM file from the previous step was uploaded. For the Explore: Metagenome Inference: PICRUSt2: parameters were 2 for Max NSTI, 1 for min reads, 1 for min Samples, none for stratified. For the MicrobiomeDB analysis, the DEseq2 was used to calculate the differential abundance. For the Microbiomeanalalyst analysis, the Marker Data Profiling was used. The parameters were 4 for minimum count, 20% prevalence in samples for the low count filter, inter-quantile range for low variance filter. In the data normalization, rarefy my data and transform data were not selected, total sum scaling (TSS) was selected for data scaling. In the alpha diversity profiling, filtered data was selected, Chao1 was for diversity measure, Mann-Whitney/Krustkal-Wallis was chosen for the statistical method. In the beta diversity, PCoA was selected for the ordination method, Bray-Curtis Index was chosen for the Distance method, PERMANOVA was chosen for the statistical method. For the clustering heatmap visualization, Euclidean method was chosen for distance measure and ward method for clustering algorithm. For the correlation analysis, the parameters were using the SECOM (Pearson) for algorithm, 100 for permutation (SparCC), 0.05 for p-value threshold, 0.3 for correlation threshold. For random forest classification, the parameters were using feature-level for taxonomy, 2000 for number of trees to grow, 15 for number of predictors to try, and randomness setting was on.

### ITS sequencing analysis

ITS sequences were pre-processed, quality filtered, and analysed using the Nephele platform (https://nephele.niaid.nih.gov/) from the National Institute of Allergy and Infectious Diseases (NIAID) Office of Cyber Infrastructure and Computational Biology (OCICB) in Bethesda, Maryland, USA [31]. The paired End ITS FASTQ files were analysed with the DADA2 ITS pipeline in Nephele with default settings. Specifically, the parameters were 4 for Truncation quality score, 5 for maximum expected errors, and 50 for minimum read length. For the MicrobiomeDB analysis, the DEseq2 was used to calculate the differential abundance.

### Statistical analysis

Kaplan-Meier analysis was performed for survival analysis, and significance between survival curves was determined by a log-rank test. Assumptions for Kaplan-Meier were checked for its appropriateness for the survival analysis, such as the subjects were independent, the entry criteria were consistent, the end point was defined consistently, the time of censoring was not related to survival, the average survival stay was constant during the course of the study, the proportional hazards assumption was reasonable, and the treatment groups were defined before data collection began. P < 0.05 is considered significantly different. Sequencing data significant analysis were done using the default parameters of the Nephele, MicrobiomeDB and microbiomeanalyst programs with p < 0.05 as significantly changed.

### Electronic supplementary material

Below is the link to the electronic supplementary material.

**Supp Fig. S1**. FastQC sequence count of each 16S rRNA reading. Number of reads for each sample were shown on the X-axis, and sample names were labelled on the left. Blue colour represents unique reads and black colour represents duplicate reads.

**Supp Fig. S2**. Mean quality scores of each 16S rRNA reading. Green colour represents forward reading sequences and yellow colour represents reverse reading.

**Supp Fig. S3**. Per sequence quality scores of each 16S rRNA reading. Each green line represents one sample’s reading.

**Supp Fig. S4**. Alpha rarefaction of forward reading. X-axis is the sequence depth and Y-axis is the Shannon index. Each line represents one sample.

**Supp Fig. S5**. Full image of the metabolic pathways identified in IL18 wild type and knockout mice using the PICRUST2 pipeline.

**Supp Fig. S6**. PCOA of IL-18 knockout and wild type mice ITS sequences. (a) the PCOA of all mice, (b) the PCOA of all mice based on genotype and cohousing status, (c) the PCOA of all mice based on time after radiation exposure. Each dot represents one faecal sample of one mouse at one time.

**Supp Table 1**. Detailed number of faecal samples collected.

**Supp Table 2**. General statistics of the 372 16S sequencing files.

**Supp Table 3**. Detailed list of significantly changed bacterial genera of the pure IL-18 knockout and wild type mice at specific time points after irradiation compared to their d0 samples respectively.

**Supp Table 4**. Detailed list of significantly changed bacteria of the pure IL-18 knockout and wild type mice.

**Supp Table 5**. Detailed list of significantly correlated bacteria genera of the pure IL-18 knockout and wild type mice.

## Data Availability

The authors declare that all the data supporting the findings of this study are provided within the manuscript, additional information file, and provided links. Raw sequenced reads generated from the Illumina platform have been deposited into the NCBI Sequence Read Archive public database, with SRA accession numbers SUB12793806(16S data) and SUB12532293 (ITS data). The reviewer links for the sequencing data are: https://dataview.ncbi.nlm.nih.gov/object/PRJNA909249?reviewer=q7enr0d1bande64ej9q7s2phk2 (16S data) and https://dataview.ncbi.nlm.nih.gov/object/PRJNA932308?reviewer=mojb5t1bojt673nf741a5o6m53 (ITS data).
