# Supplementary figures and images for: The gut microbiome changes in wild type and IL-18 knockout mice after 9.0 Gy total body irradiation
Source: Anim Microbiome. 2023 Sep 7;5:42. doi: 10.1186/s42523-023-00262-8 (PMC10485964; doi:10.1186/s42523-023-00262-8)

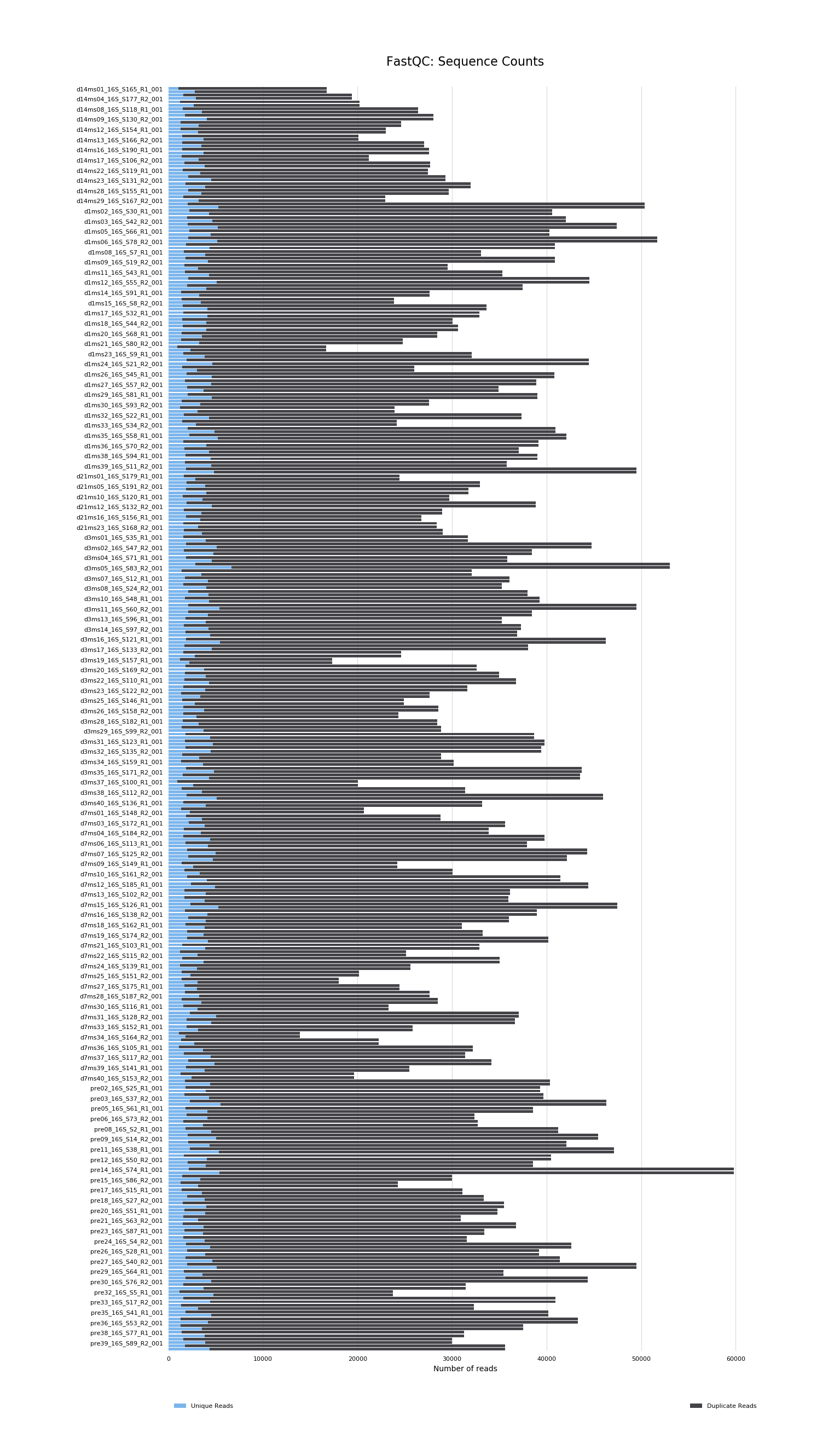

Supplement: Supplementary file 1 — Supp Fig. S1. FastQC sequence count of each 16S rRNA reading. Number of reads for each sample were shown on the X-axis, and sample names were labelled on the left. Blue colour represents unique reads and black colour represents duplicate reads. [file 42523_2023_262_MOESM1_ESM.png]

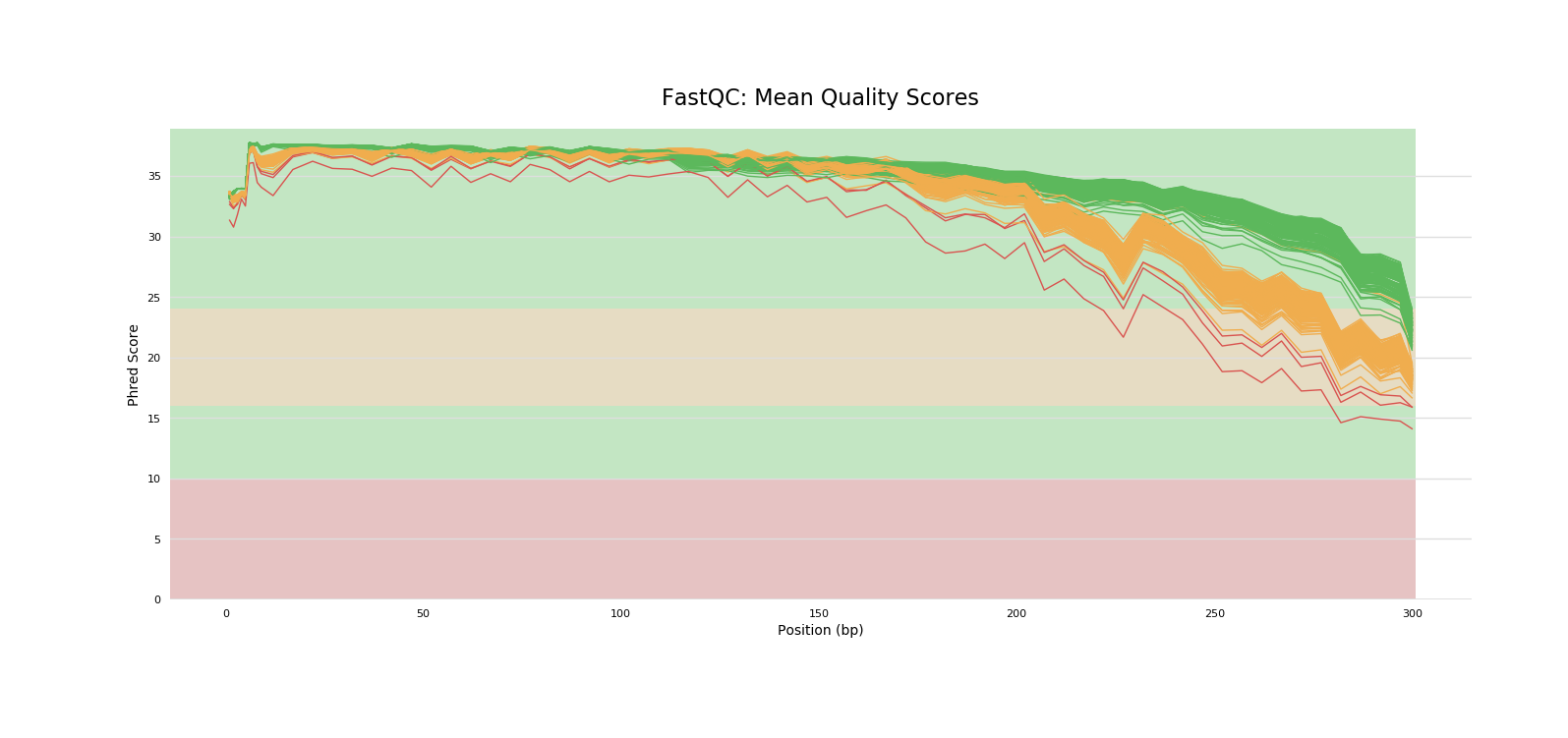

Supplement: Supplementary file 2 — Supp Fig. S2. Mean quality scores of each 16S rRNA reading. Green colour represents forward reading sequences and yellow colour represents reverse reading. [file 42523_2023_262_MOESM2_ESM.png]

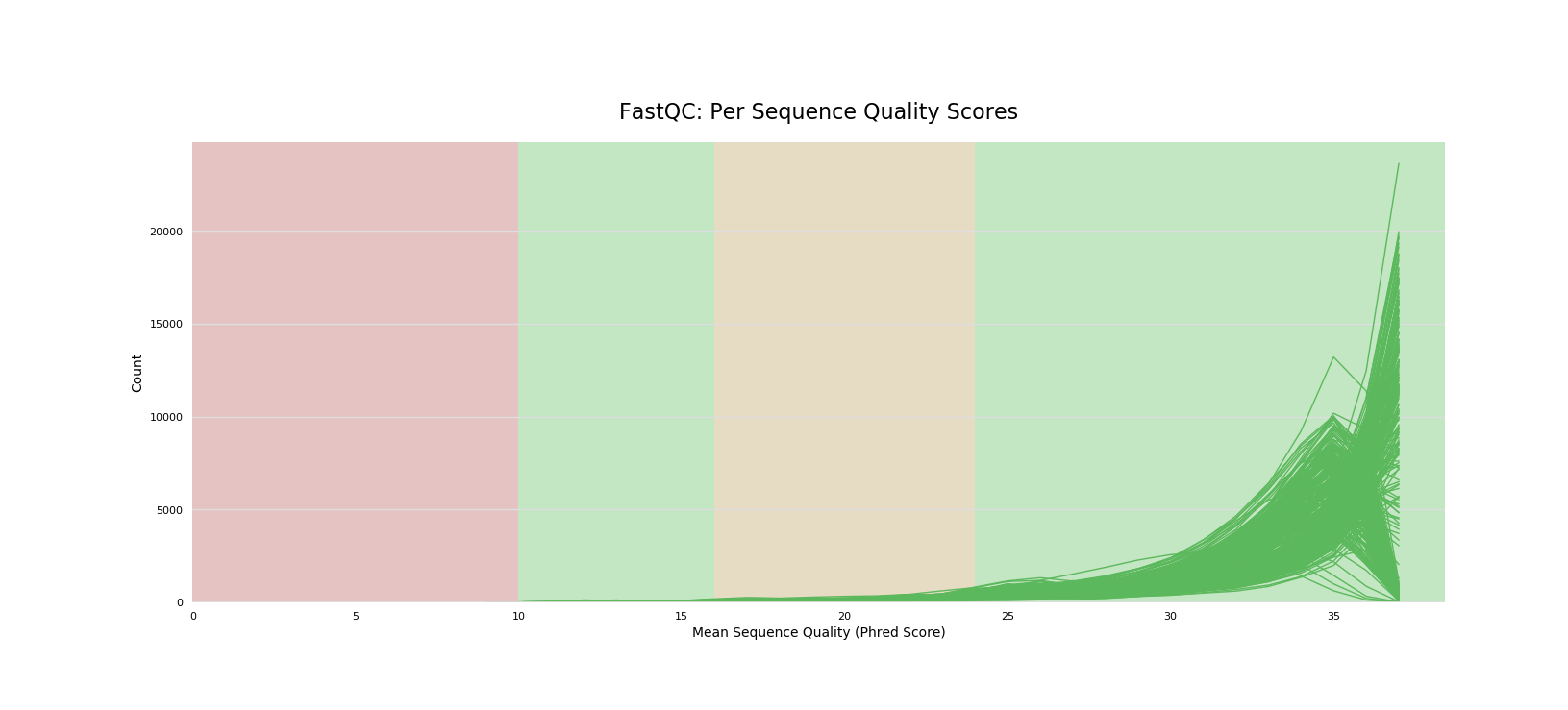

Supplement: Supplementary file 3 — Supp Fig. S3. Per sequence quality scores of each 16S rRNA reading. Each green line represents one sample’s reading. [file 42523_2023_262_MOESM3_ESM.png]

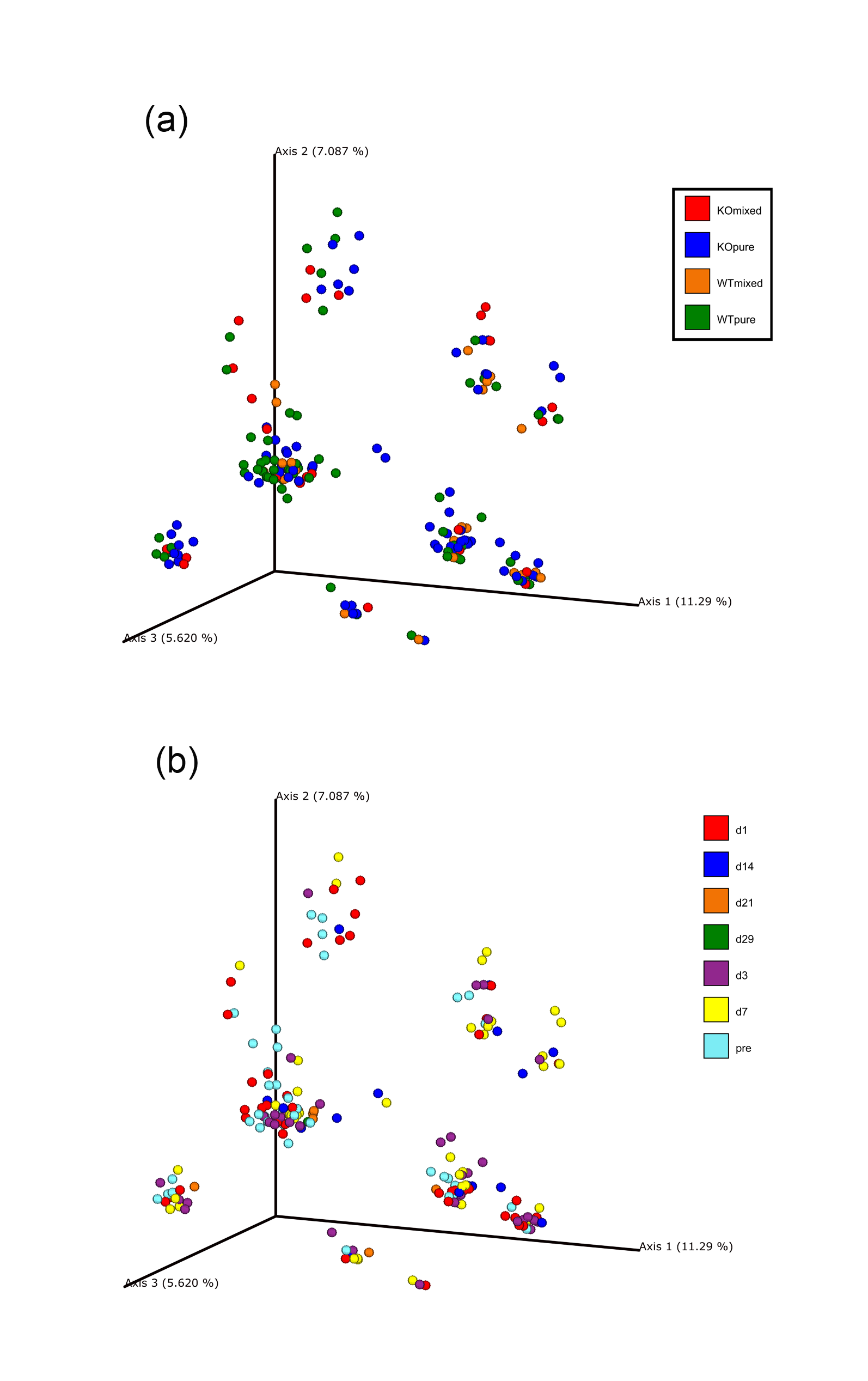

Supplement: Supplementary file 6 — Supp Fig. S6. PCOA of IL-18 knockout and wild type mice ITS sequences. (a) the PCOA of all mice, (b) the PCOA of all mice based on genotype and cohousing status, (c) the PCOA of all mice based on time after radiation exposure. Each dot represents one faecal sample of one mouse at one time. [file 42523_2023_262_MOESM6_ESM.png]
