# Supplementary material for: The gut microbiome changes in wild type and IL-18 knockout mice after 9.0 Gy total body irradiation
Source: Anim Microbiome. 2023 Sep 7;5:42. doi: 10.1186/s42523-023-00262-8 (PMC10485964; doi:10.1186/s42523-023-00262-8)
Supplement: Supplementary file 4 — Supp Fig. S4. Alpha rarefaction of forward reading. X-axis is the sequence depth and Y-axis is the Shannon index. Each line represents one sample. [file 42523_2023_262_MOESM4_ESM.pdf]

(http://qiime2.org/)

Alpha rarefaction

The following metadata columns have been omitted because they didn't contain categorical data, or the column consisted only of missing values: **Description**

Download CSV (shannon.csv)

Metric

shannon

Sample Metadata Column

forward-absolute-filepath

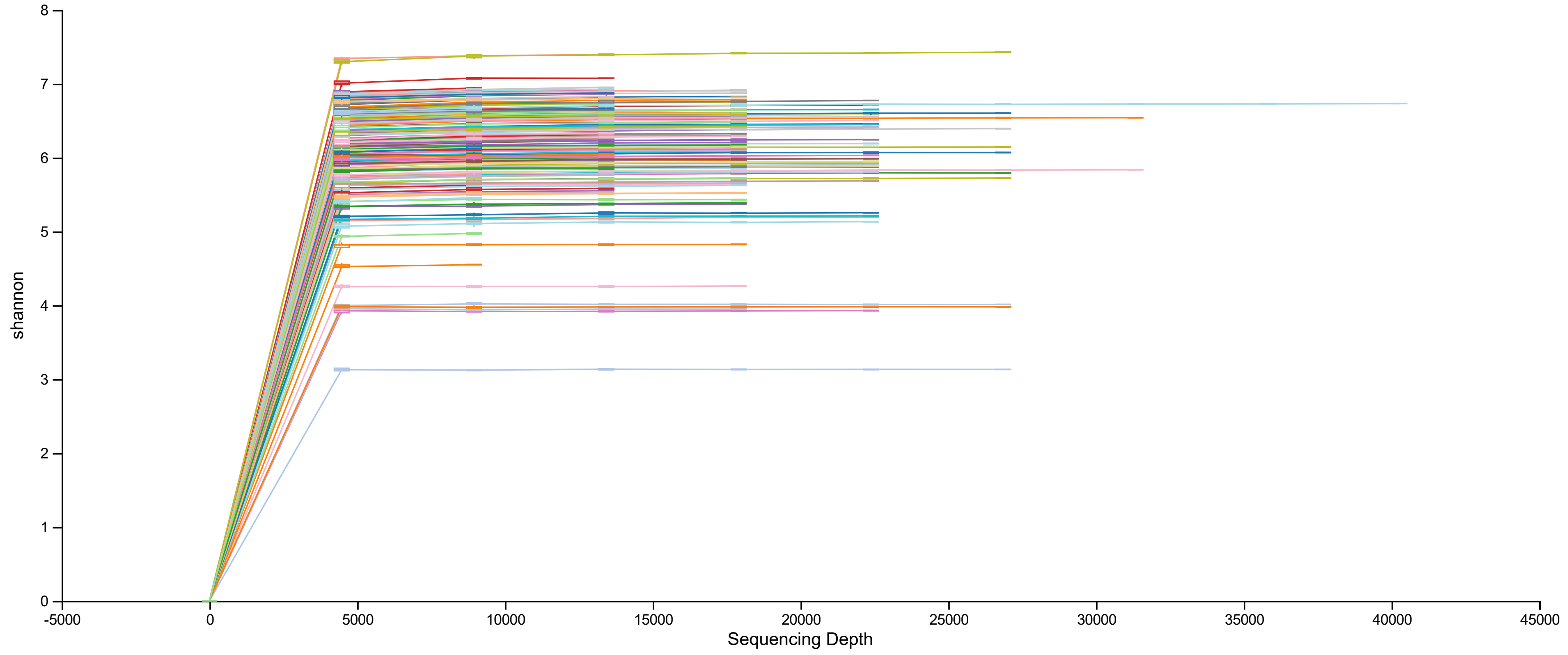
shannon
Sequencing Depth
0
1
2
3
4
5
6
7
8
-5000
0
5000
10000
15000
20000
25000
30000
35000
40000
45000

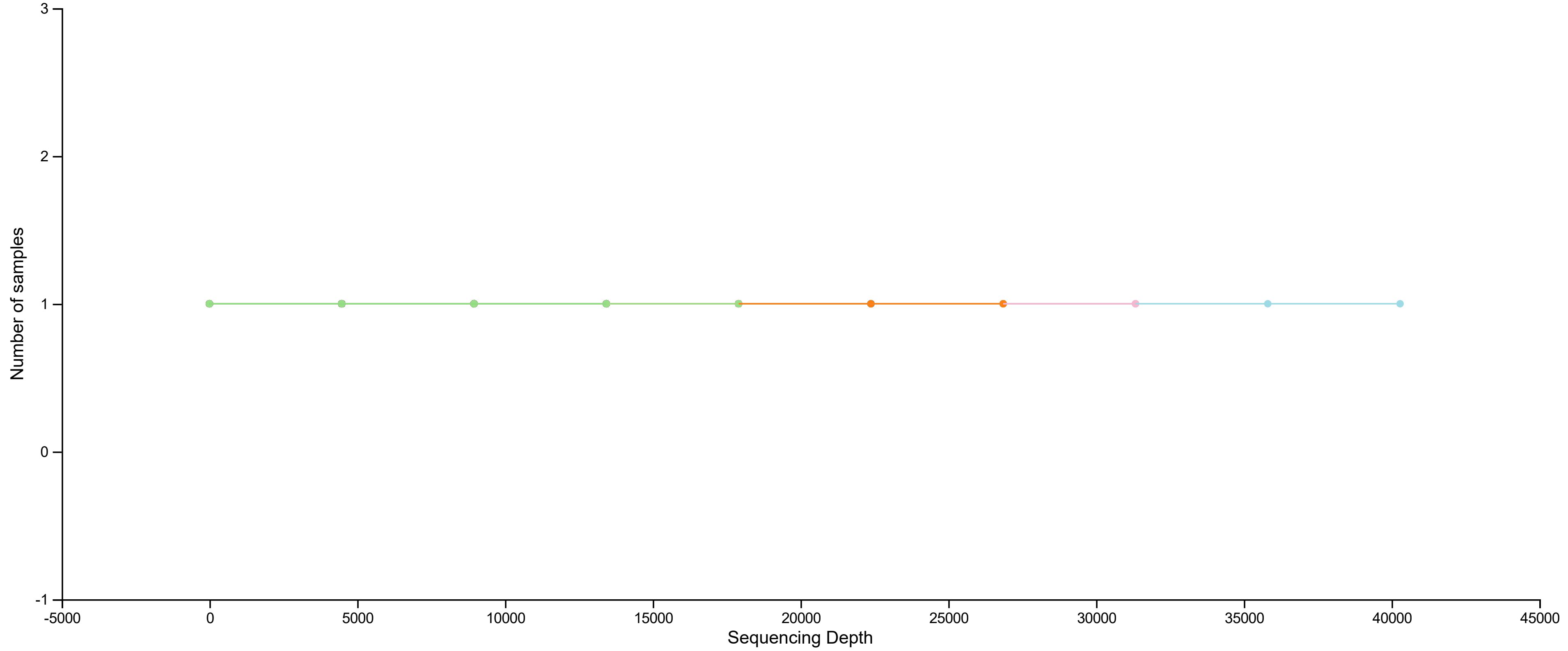
Number of samples
Sequencing Depth
-1
0
1
2
3
-5000
0
5000
10000
15000
20000
25000
30000
35000
40000
45000

Help

Select All

/nephele_data/inputs/d14ms01_16S_S165_R1_001.fastq
/nephele_data/inputs/d14ms04_16S_S177_R1_001.fastq
/nephele_data/inputs/d14ms05_16S_S189_R1_001.fastq
/nephele_data/inputs/d14ms08_16S_S118_R1_001.fastq
/nephele_data/inputs/d14ms09_16S_S130_R1_001.fastq
/nephele_data/inputs/d14ms10_16S_S142_R1_001.fastq
/nephele_data/inputs/d14ms12_16S_S154_R1_001.fastq
/nephele_data/inputs/d14ms13_16S_S166_R1_001.fastq
/nephele_data/inputs/d14ms14_16S_S178_R1_001.fastq
/nephele_data/inputs/d14ms16_16S_S190_R1_001.fastq
/nephele_data/inputs/d14ms17_16S_S106_R1_001.fastq
/nephele_data/inputs/d14ms20_16S_S107_R1_001.fastq
/nephele_data/inputs/d14ms22_16S_S119_R1_001.fastq
/nephele_data/inputs/d14ms23_16S_S131_R1_001.fastq
/nephele_data/inputs/d14ms24_16S_S143_R1_001.fastq
/nephele_data/inputs/d14ms28_16S_S155_R1_001.fastq
/nephele_data/inputs/d14ms29_16S_S167_R1_001.fastq
/nephele_data/inputs/d1ms01_16S_S18_R1_001.fastq
/nephele_data/inputs/d1ms02_16S_S30_R1_001.fastq
/nephele_data/inputs/d1ms03_16S_S42_R1_001.fastq
/nephele_data/inputs/d1ms04_16S_S54_R1_001.fastq
/nephele_data/inputs/d1ms05_16S_S66_R1_001.fastq
/nephele_data/inputs/d1ms06_16S_S78_R1_001.fastq
/nephele_data/inputs/d1ms07_16S_S90_R1_001.fastq
/nephele_data/inputs/d1ms08_16S_S7_R1_001.fastq
/nephele_data/inputs/d1ms09_16S_S19_R1_001.fastq
/nephele_data/inputs/d1ms10_16S_S31_R1_001.fastq
/nephele_data/inputs/d1ms11_16S_S43_R1_001.fastq
/nephele_data/inputs/d1ms12_16S_S55_R1_001.fastq
/nephele_data/inputs/d1ms13_16S_S67_R1_001.fastq
/nephele_data/inputs/d1ms14_16S_S91_R1_001.fastq
/nephele_data/inputs/d1ms15_16S_S8_R1_001.fastq
/nephele_data/inputs/d1ms16_16S_S20_R1_001.fastq
/nephele_data/inputs/d1ms17_16S_S32_R1_001.fastq
/nephele_data/inputs/d1ms18_16S_S44_R1_001.fastq
/nephele_data/inputs/d1ms19_16S_S56_R1_001.fastq
/nephele_data/inputs/d1ms20_16S_S68_R1_001.fastq
/nephele_data/inputs/d1ms21_16S_S80_R1_001.fastq
/nephele_data/inputs/d1ms22_16S_S92_R1_001.fastq
/nephele_data/inputs/d1ms23_16S_S9_R1_001.fastq
/nephele_data/inputs/d1ms24_16S_S21_R1_001.fastq
/nephele_data/inputs/d1ms25_16S_S33_R1_001.fastq
/nephele_data/inputs/d1ms26_16S_S45_R1_001.fastq
/nephele_data/inputs/d1ms27_16S_S57_R1_001.fastq
/nephele_data/inputs/d1ms28_16S_S69_R1_001.fastq
